# Unlocked yet untapped: The ubiquitous smartphone and utilization of emergency medical identification technology in the care of the injured patient^☆^

**DOI:** 10.1016/j.sopen.2020.03.001

**Published:** 2020-04-12

**Authors:** Michael A. Vella, Howard Li, Patrick M. Reilly, Shariq S. Raza

**Affiliations:** a Division of Traumatology, Surgical Critical Care and Emergency Surgery, Perelman School of Medicine at the University of Pennsylvania, Philadelphia, PA, USA; b Division of Acute Care Surgery and Trauma, University of Rochester School of Medicine and Dentistry, Rochester, NY, USA

## Abstract

**Background:**

Smartphones allow users to store health and identification information that is accessible without a passcode—conceivably invaluable information for care of unresponsive trauma patients. We sought to characterize the use of smartphone emergency medical identification applications and hypothesized that these are infrequently used but positively perceived.

**Methods:**

We surveyed a convenience sample of adult trauma patients/family members (nonproviders) and providers from an urban Level I trauma center during July 2018 on their demographics and smartphone emergency medical identification application usage. Descriptive and chi-square/Fisher exact analyses were performed to characterize the use of smartphone emergency medical identification applications and compare groups.

**Results:**

338 subjects participated; most were female (52%) with median age of 36 (29–48). 182 (54%) were providers and 306 (91%) owned smartphones. 157 (51%) owners were aware smartphone emergency medical identification existed, but only 94 (31%) used it. 123 providers encountered unresponsive patients with smartphones, but only 26 (21%) queried smartphone emergency medical identification, with 19 (73%) finding smartphone emergency medical identification helpful. All 8 (100%) nonproviders who reported to have had their smartphone emergency medical identification queried believed it was beneficial. There were no differences between groups in smartphone emergency medical identification awareness and utilization.

**Conclusion:**

Smartphone emergency medical identification technology is underused despite its potential benefits. Future work should focus on improving education to use this technology in trauma care.

## 1 BACKGROUND

The smartphone is a technology that “combines mobile communication and computation in a handheld device, facilitating mobile computing at the point of care” [1]. Over 60% of Americans and 80% of those ages 18–49 own smartphones [2]. Fifty percent of owners use their devices for health information, and at least 20% of owners have downloaded applications, defined as pieces of software designed specifically for a mobile device for a specific purpose [2,3]. Over 40,000 health-related smartphone applications exist, the most common relating to exercise and weight loss [3]. Unfortunately, it can sometimes be difficult to determine application quality, with one study finding that only 6.9% of all medical-related applications from the iTunes App Store (Apple, Inc) were clinically relevant [4].

As of 2012, at least 19 applications had been developed to allow owners to store important health information on their phones [3,5]. Both major smartphone operating system manufacturers including Apple (iOS) and Google (Android) offer a built-in application that allows users to store identification and emergency contact information, age, medical conditions, medications, allergies, blood type, organ donation status, and other health-related notes [6,7]. These smartphone emergency medical identification (SEMID) applications also give owners the option to let this information bypass the passcode lock protection, hence allowing accessibility by health care providers through a locked screen. These features could potentially be useful in situations where trauma patients are unable to provide medical history, particularly if that information could be relevant to direct immediate management. Furthermore, there could be other intangible yet significant positive impacts of earlier identification of unidentified trauma patients, from earlier family notification affecting overall patient and family satisfaction to potential for increased organ donation in case of dire circumstances by reducing time to locate next of kin. In our institution alone in 2017, 6% of trauma patients arrived with Glasgow Coma Scale (GCS) scores of ≤ 8 and would have been unable to provide important medical information. Additionally, 10% arrived with GCS scores of ≤ 12, with a significant number of these patients likely unable to provide a useful or coherent history (unpublished institutional data).

Most studies evaluating the use of smartphones in medicine have focused on applications used to enhance communication and medical decision making, educate patients and providers, and track disease processes over time [1,3,[8], [9], [10], [11]]. Little is known about awareness and utilization of the free and relatively simple-to-use SEMID applications, especially in the trauma setting. We sought to characterize the awareness and use of SEMID applications among trauma patients, family members, and providers and hypothesized that these functions are infrequently used but positively perceived.

## 2 MATERIALS AND METHODS

After University of Pennsylvania Institutional Review Board approval, we identified a convenience sample of adult (≥ 18 years old) trauma patients, patient family members, and multidisciplinary providers involved in the care of trauma patients from an urban level I trauma center during July 2018. A trained undergraduate student (HL) surveyed these subjects in the emergency department, general trauma floor, and trauma/surgical ICU over the 4-week study period during normal business hours (8:00 am to 4:00 pm). Surveys were completed on an electronic tablet. Only subjects able to functionally complete study questionnaires at that point of their evaluation or admission were asked to participate. Subjects completed a 30-question survey detailing basic demographic information, smartphone ownership, and personal awareness and utilization of SEMID applications. Patients and family members were combined into a single group representative of and termed *nonproviders*. These nonproviders were then compared to providers. In addition, providers were asked about use of SEMID applications for patient care in their own practice. At the completion of each survey, study personnel offered assistance in setting-up SEMID applications. Mann-Whitney *U* test was used to compare medians between groups. Chi-square and Fisher exact analyses were performed to compare categorical variables where appropriate. SPSS (IBM Corp, Armonk, NY) was used for all statistical analysis.

## 3 RESULTS

### 3.1 Overall Participant Characteristics and SEMID Application Awareness

During the month-long study period, the trauma service evaluated 255 contacts (admitted and nonadmitted trauma activations and consultations), with 84 patients ultimately being admitted to the trauma service. Three hundred and forty-two subjects were asked to participate, and 338 (99%) agreed. Participants were mostly female (52%), with a median age of 36 (29–48). One hundred eighty-two (54%) were providers, and 306 (91%) owned a smartphone. Two hundred nine (68%) owned an Apple iOS–based iPhone, 93 (30%) owned a Google Android operating system–based phone, and 4 (1%) owned another device. One hundred and fifty-seven (51%) smartphone owners were aware of SEMID applications; 94 (31% of all owners and 60% of those aware of the technology) had set up these functions in their phones.

**Fig. 1:**
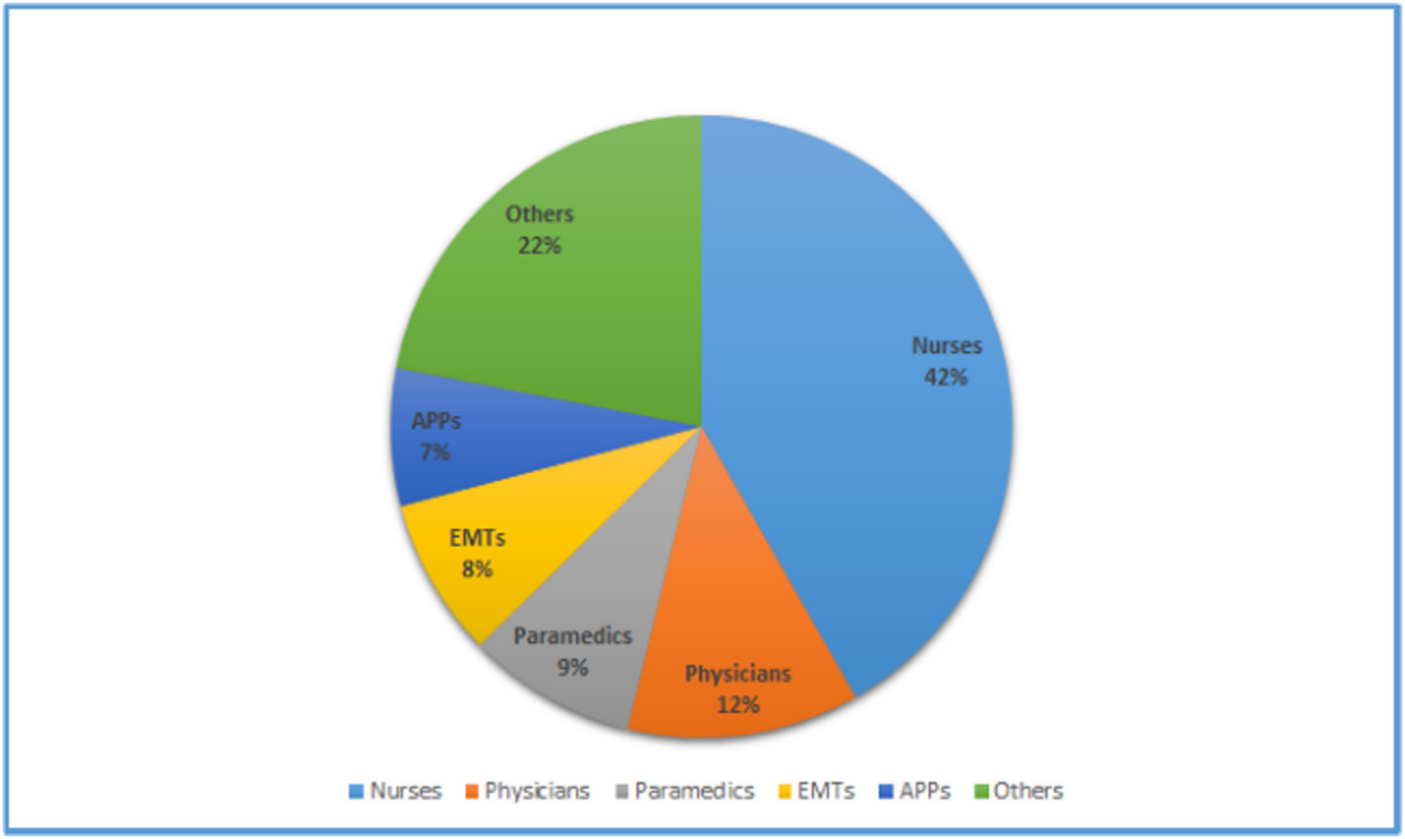
Provider make-up (*n* = 182). EMT = emergency medical technician; APP = advanced practice provider; Others = respiratory therapists, patient care technicians, firefighters, chaplains, police officers, radiology technicians, and other professionals. Median practice time 7 (4–16) years.

The provider group was made up of 76 (42%) nurses, 22 (12%) physicians, 16 (9%) paramedics, 15 (8%) emergency medical technicians, 13 (7%) advanced practice providers, 5 (3%) respiratory therapists, 5 (3%) patient care technicians, 3 (2%) firefighters, 2 (1%) chaplains, 1 (0.6%) police officer, 1 (0.6%) radiology technician, and 23 (13%) other professionals with a median practice time/job experience of 7 (4–16) years (Fig. 1).

**Fig. 2:**
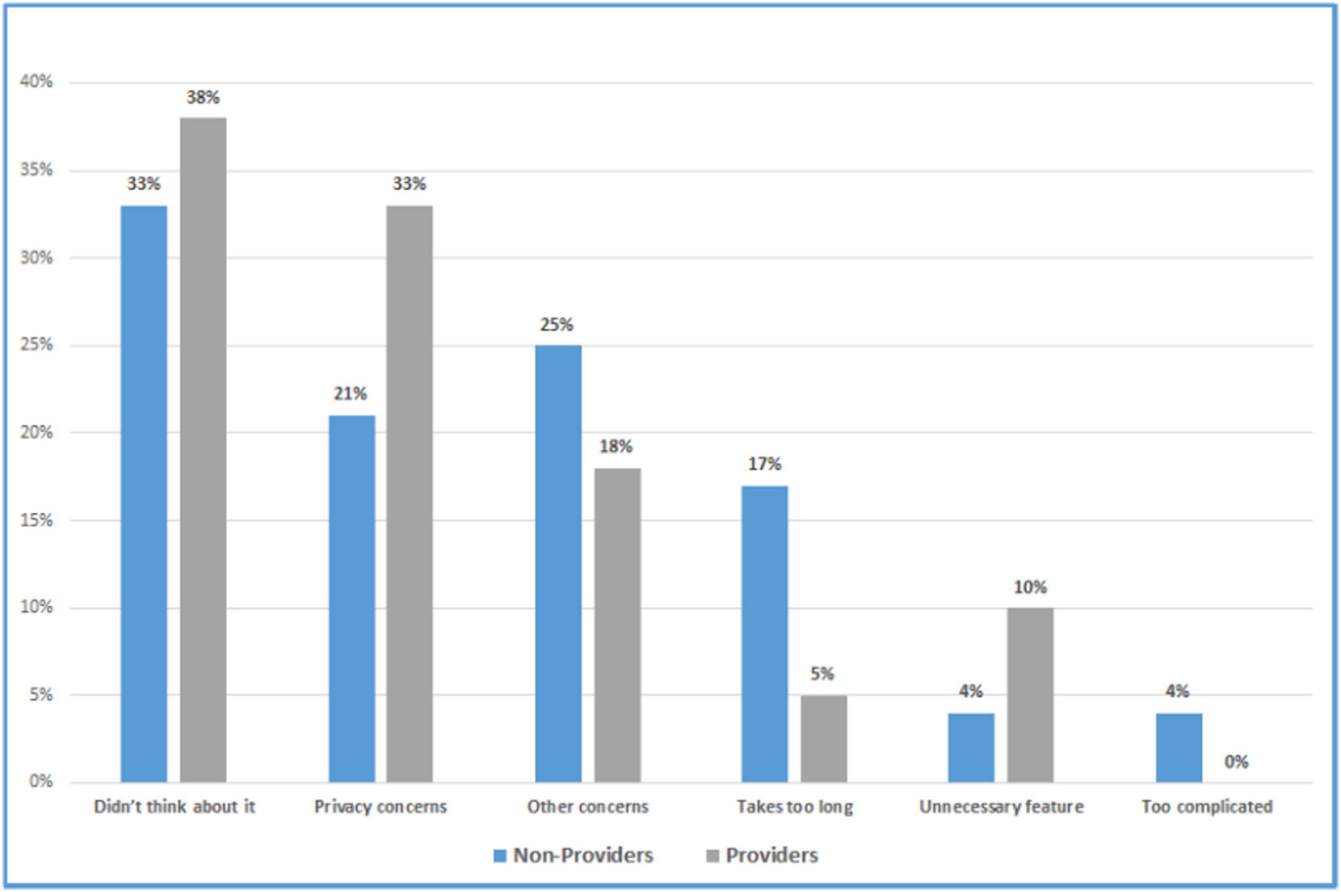
Reasons cited for not setting up SEMID feature. *Of smartphone owners aware of the feature but do not use it. All *P* > .05. Participants were able to select > 1 response.

Sixty-three (40%) smartphone owners were aware of SEMID applications but did not use them. The reasons cited for not setting up SEMID features did not differ between nonprovider and provider groups and included “didn't think about it” (33% vs 38%, *P* = .79), “privacy concerns” (21% vs 33%, *P* = .38), “other concerns” (25% vs 18%, *P* = .54), “takes too long” (17% vs 5%, *P* = .19), “not needed” (4% vs 10%, *P* = .64), and “too complicated” (4% vs 0%, *P* = .38) (Fig. 2).

### 3.2 Utilization of SEMID in Providers and Nonproviders for Personal Medical Care

All 8 (100%) nonproviders who reported having their SEMID application queried in a medical situation believed it was beneficial for their care. One hundred seventy-five (97%) provider smartphone owners believed that SEMID applications could be useful for their own care in the event of an emergency, although only 1 (0.5%) provider previously had their SEMID application queried. Of smartphone nonowners, 26 (81%) believed SEMID applications would be helpful in an emergency, and 24 (75%) would set up these features if they owned a smartphone.

### 3.3 Utilization of SEMID in Providers as Caregivers

Of the 123 (68%) providers who had encountered an unresponsive patient with a smartphone, only 26 (21%) queried patient's SEMID application. Nineteen (73%) of those providers found it helpful for that patient's care. Of the 163 providers who have not queried a phone **or** have queried a phone but have not found it useful, 159 (98%) believed that SEMID could be helpful in the right clinical circumstance. One hundred seventy-one (95%) providers who own smartphones believed that more providers would query and use this function if education were provided. Twenty-three (13%) providers reported that they were unaware of policies regarding what to do with unresponsive patient's cellphone.

All 22 (100%) physician providers (median age 32 [28–34], 36% female) owned smartphones. Thirteen (59%) were aware of SEMID applications. However, only 1 (5%) physician had queried SEMID for their patient's care. Despite this, 21 (95%) believed these applications could be helpful in an emergency.

### 3.4 Comparison of SEMID Awareness Between Providers and Nonproviders

When compared to nonproviders, providers were younger (33 [28–43] years vs 42 [30–53] years, *P* < .001), more likely to be female (60% vs 45%, *P* = .005), and more likely to own a smartphone (99% vs 81%, *P* < .001). Provider smartphone owners who did not use SEMID features were more likely to agree that these features could be helpful in a personal emergency (96% vs 70%, *P* < .001). There were no differences between providers and nonproviders with respect to SEMID awareness (54% vs 48%, *P* = .280), SEMID utilization among smartphone owners (32% vs 29%, *P* = .464), or utilization among those aware of the technology (60% vs 60%, *P* = .980). One hundred thirteen (90%) and 164 (91%) of nonprovider and provider smartphone owners, respectively, believed that SEMID setup and utilization would increase among patients and providers if education were provided (*P* = .675) (Table).

**Table:** Comparing use of SEMID technology between nonproviders and providers

*Variable*	*Nonproviders* *(*n *= 156)*	*Providers* *(*n *= 182)*	P *value*
Age	42[30–53]	33[28–43]	< .001
Sex (female)	70 (45%)	110 (60%)	.005
Own smartphone Aware of SEMID feature Have SEMID set up on phone	126 (81%) 60 (48%⁎) 36 (29%⁎, 60%†)	180 (99%) 97 (54%⁎) 58 (32%⁎, 60%†)	< .001 .280 .464⁎, .980†
Believe SEMID features could be helpful for their own care in an emergency‡	71 (79%)	117 (96%)	< .001
Believe more education will increase SEMID utilization	113 (90%⁎)	164 (91%⁎)	.675

Data for nonparametric continuous variables expressed as median (interquartile range); categorical values expressed as *n* (%).

⁎ Of those who own a smartphone.

† Of those aware of the technology.

‡ Of those who own smartphones but do not use SEMID features.

## 4 DISCUSSION

Despite high rates of smartphone ownership in both provider and nonprovider groups, both populations reported relatively low awareness and utilization of SEMID technology for patient care and personal use. Of participants who did not use SEMID applications, providers were more likely to agree that SEMID technology could be helpful in the event of a personal medical emergency compared with nonproviders. Both groups believed that education would improve utilization of SEMID applications. To our knowledge, this is the first study to describe the awareness and utilization of SEMID applications in the trauma population.

Electronic personal health records (PHRs) allow for the organization and storage of personal health information that is, at least in part, accessed and managed by the patient [5,12,13]. In the early 2000s, only about 2% of patients had personal health information stored on computers; today, about 70 million Americans are thought to have access to PHR, the use of which has been increasing since the development of mobile devices and applications [5,14]. Initially, “mobile” PHR (mPHR) information was stored on USB drives or CDs and, in the event of an emergency, typically required external devices to access the data [5]. Today, there are at least 19 smartphone applications that vary in function and price and allow patients to store important medical information [3,5]. PHR applications have been shown to improve outcomes in certain chronic medical conditions and to assist with medication administration [13].

Only about 2% of smartphone mPHR applications are designed specifically for use in emergency situations [15]. These SEMID or “in case of emergency” applications have been built into the operating systems of multiple smartphone devices and were designed specifically for third-party access in the event that a patient cannot provide a medical history [[5], [6], [7]]. The majority of literature evaluating smartphone applications does not address SEMID features specifically, but our results can be compared to studies that have evaluated mPHR and other smartphone applications more broadly. In a study of 3,165 patients from the 2013 Health Information National Trends Survey, for example, 6% of participants reported using a mobile application for health information exchange [16]. In contrast, 29% of nonproviders and 32% of providers (31% of all smartphone owners) reported using personal SEMID applications in the current study, a difference that may be explained by easy accessibility and the relative simplicity of SEMID application setup and use. Interestingly, SEMID awareness and personal utilization were low overall and did not differ between nonproviders and providers despite high smartphone ownership in both groups (81% and 99%, respectively) and a robust literature showing the widespread use of health care applications by providers (mainly physicians) for patient care [1,3,[8], [9], [10],^15^,^17^,^18^]. In fact, 87% of physicians in 1 survey reported that smartphones are essential to their practice [11]. We did find that provider smartphone owners who did not use SEMID applications were more likely to find them potentially beneficial for their own personal care compared to their nonprovider counterparts.

In addition to personal use, we also found that providers reported a relatively low utilization of SEMID features for patient care. In our study, only 21% of providers report querying a smartphone (including 1/22 physicians) despite 68% having encountered an unresponsive patient with a smartphone. However, the majority of providers, including 95% of physicians, report that SEMID technology could be beneficial for patient care. In a national survey of 865 US physicians, 64% had never used an electronic PHR for patient care, 42% agreed that they would be willing to use electronic PHRs despite limited experience with the technology, and 53% agreed that electronic PHRs could improve the quality of care of their patients [19]. Our results suggest that providers are likely to find applications designed for emergency use more beneficial.

In the current study, 40% of smartphone owners in both groups were aware of SEMID applications yet did not set up these features on their phone. “Not thinking about it” and “privacy concerns” were common reasons for not setting up SEMID applications in both nonprovider and provider participants, consistent with prior work evaluating health applications that store diagnostic information [5,16,17]. In a 2015 study by Krebs et al. of 1,604 mobile phone users in the United States, 42% had never downloaded a health application [2]. The most common reasons for nonuse were lack of interest (27%), high cost (23%), lack of trust in applications collecting personal data (15%), concern about excessive data use (13%), and belief that they did not need a health application (11%). Only 34% of owners reported they trusted their app's data security. In stark contrast to other electronic medical record platforms, SEMID applications are specifically designed to offer owners the option to allow open third-party access to personal information and afford users complete control over what and how much personal, medical, and contact information is available for their own emergent care. In general, information from SEMID applications is not cloud based and theoretically not accessible without a device.

Perhaps the most important finding of the current study is the belief among nonproviders and providers alike (even nonowners) that SEMID technology could prove beneficial in emergency situations. Eight nonproviders reported having their own SEMID application queried in an emergency, and all believed that it was useful for their care. Seventy-three percent of providers who have queried a smartphone for patient information found it beneficial for patient care, and the majority of those who did not still believed that it could be useful in a different situation. The majority of nonproviders (71%) and providers (96%) reported that SEMID applications could be useful for their own care, and the majority (73%) of non–smartphone owners would set up such a feature if they did own a smartphone. A 2013 study of 152 adult inpatients from a single urban institution in California found that 56% of patients brought smartphones with them to the hospital, and 95% used it during their stay [20]. Perhaps inpatient admission provides an opportunity for providers to educate patients on the importance of SEMID features as well as assist in setup. Because both groups in the current study believed that education would improve SEMID utilization, supported by the fact that SEMID utilization was higher among those aware of the feature (60%), our results suggest that formal education of nonproviders and providers alike would be beneficial. The finding that 13% of providers were unaware of policies regarding disposition of smartphones found on unresponsive trauma patients also suggests a role for more formal policy development and education from an administrative standpoint. The current policy at our urban Level 1 trauma center is to secure the phone along with patient's other belongings, without any specific process or requirement to query the phones for SEMID or “in case of emergency” contacts. Since gathering these study data, we have embarked on a performance improvement project to actively query smartphones of all unresponsive trauma patients for SEMID information.

The benefits of SEMID applications extend beyond those involved in the care of patients at a single trauma center. Although not specifically addressed in the current study, smartphone applications have been developed for use in mass casualty incidents, such as terrorist attacks and natural disasters [21]. Awareness and utilization of SEMID applications could be particularly useful in these events as well, where immediate access to demographic and medical information is paramount. Indeed, anecdotal evidence suggests adolescents' and millennials' increasing proclivity to carry only a smartphone on their person to serve multiple functions, including communication and point-of-sale electronic payments. Perhaps there are even further potential benefits to SEMID technology that are undefined as of yet—from improved patient satisfaction by earlier family notification to possible increase in potential organ donation via earlier identification of unidentified patients with devastating injuries.

We acknowledge the limitations of this study. It is a convenience sample of nonproviders and providers, which introduces potential selection bias. Because of relatively small sample sizes in each group, we did not compare SEMID awareness and utilization among all provider types. In addition, all participants hailed from a single institution and geographical region, and results may not be generalizable to other nonprovider populations, providers who do not take care of trauma patients, or other institutions.

We would be remiss if we did not at least entertain the flaw in the premise that possession of the smartphone assumes ownership. Further work is needed to clarify the validity of these concerns.

Finally, although our study gauged awareness and utilization of SEMID applications, we did not query individual smartphones nor did we correlate SEMID awareness and utilization with specific patient and provider demographics/characteristics. The purpose of this pilot study was to determine overall awareness and utilization of these functions in a sample representative of a general trauma population, with future work aimed at determining if and how SEMID applications can be used to better patient care.

In conclusion, SEMID technology is severely underused among trauma patients/family members and providers despite its perceived benefits. Use is higher among those aware of these applications, suggesting that education may improve rates of utilization among nonproviders and providers alike. Future work should focus on how to best use this technology in the care of trauma patients and determining the effects of these applications on trauma patient outcomes.

## Author Contributions

**MAV:** study design, data analysis, manuscript drafting, manuscript editing

**HL:** study design, data acquisition, data analysis, manuscript editing

**PMR:** data acquisition, data analysis, manuscript editing

**SSR:** study design, data analysis, manuscript editing

## Conflicts of Interest

The authors declare no conflicts of interest.

## Sources of Funding

The authors declare no sources of funding.
